# 3DI: A novel ion composition and three-dimensional velocity analyzer for the topside ionosphere

**DOI:** 10.1038/s41598-020-64407-4

**Published:** 2020-05-14

**Authors:** Keiichi Ogasawara, Don E. George, Jerry Goldstein, Kyoung-Joo Hwang, Yukitoshi Nishimura, David A. Ruggles, Jason L. Stange

**Affiliations:** 1 Southwest Research Institute, San Antonio, TX 78238 USA; 2 Boston University, Boston, MA 02215 USA; 3 The University of Texas at San Antonio, 1 UTSA circle, San Antonio, TX 78249 USA

**Keywords:** Magnetospheric physics, Astronomical instrumentation

## Abstract

A new ion composition and three-dimensional velocity analyzer, 3-Dimensional ion velocity and mass Imager (3DI), measures 3D velocity distribution functions (VDFs) for each major ion species in Earth’s topside ionosphere. The 3DI instrument is composed of a miniaturized electrostatic analyzer (ESA) and a deflector, backed by a static, magnet-based, mass spectrometer. We have developed a micro-pixel read-out anode technique that significantly saves power in the particle detection system, and integrated it into an imaging microchannel plate (MCP). We tested the ESA and deflector, magnet-based mass spectrometer, and anode in the laboratory to demonstrate the 3DI prototype’s performance. We have applied numerical calculations to evaluate and discuss 3DI’s performance and dynamic range. Due to complexities associated with imaging 3D distribution functions during fast spacecraft motion, we also discuss the operation strategy for 3DI to capture and resolve the VDF within the field of view. Once applied to flight investigations, the 3DI observations will be extremely useful in identifying ionosphere composition, mass-dependent ion transport such as upflows, and mass-dependent ion heating. Furthermore, the precise measurement of non-thermal plasma VDFs provides information to improve ionospheric environment modeling and ground-based radar observations.

## Introduction

We report a new three-dimensional (3D) velocity and composition analyzer, 3-Dimensional ion velocity and mass Imager (3DI), which resolves mass dependent fast flows and non-thermal features in the Earth’s topside ionosphere. 3DI measures flow velocities, fluxes, and 3D velocity distribution functions (VDFs) for major ion species. The 3DI instrument is composed of a miniaturized electrostatic analyzer (ESA); a deflector; and a static, magnet-based, mass spectrometer optimized to measure cold plasma when the spacecraft (S/C) ram speed dominates the bulk plasma speed. For the particle detection, a 2-dimensional imaging microchannel plate (MCP) is used with a novel micro-pixel read-out anode technique that can significantly reduce required power.

Current state-of-the-art instruments that cover this energy range are the Retarding Potential Analyzer (RPA), the Ion Drift Meter (IDM), ESAs, and hemispherical electrostatic analyzers (HEA). RPAs have a long, successful history of providing *in-situ* diagnostics of thermal ion spectra in Earth’s topside ionosphere^1,2^. RPAs produce a planar electric potential barrier to incoming ions. The potential varies by sweeping voltages that are applied to a set of grids. Ions with energies above the barrier are collected by a plate at the back of the instrument and measured as a current. When coupled with S/C velocity and attitude knowledge, current-voltage (I-V) curves provide ion flow speed, temperature, and density under the assumption of isotropic Maxwellian VDFs. Ion drift directions for the cross-track component can be measured using multiple sensor heads, or a segmented collector plate (measuring the current ratios among the segments). Such an instrument focusing on the ion drift direction is sometimes referred to as IDM. Due to the difference in ram energy of ions with different masses, the signatures detected by RPA voltage sweep is mass dependent. Thus, fractional compositions can be deduced from this specific response, assuming all species flow into the sensor with the same bulk speed. However, since current-voltage curves for all species are convoluted in one spectrum, it is difficult to separate non-Maxwellian VDFs and ion concentration effects by on-board momentum calculations or curve fittings. The mixture of species or hot-and-cold components in the plasma also affects IDM measurements. Thus, RPAs and IDMs usually require assumptions of one ion species (mostly O^+^ or H^+^, depending on altitude and geomagnetic activity) dominance and isotropic Maxwellian VDFs when deducing plasma parameters. Assumption of O^+^ dominated ionosphere reduces the accuracy of the measurement when H^+^ or He^+^ ions represent more than 20% of the ambient plasma^3^. Under smaller-scale height situations (e.g., night side), the H^+^ ions do become a dominant species in the ionosphere^4^. It is also known that the heating in the fast relative flows reduces O^+^ and increases NO^+^ within fast-flow channels^5^. A modeling shows that the NO^+^ ion population becomes the dominant ion species in the flow channel at <300 km, and exceed O^+^ ions even at higher (>600 km) altitudes under a strong perpendicular field^6^. In addition to the abundance anomaly, non-isotropic, non-Maxwellian distributions are very common during strong heating or acceleration, and under frequent ion-neutral interactions. Moreover, it is difficult for IDM to deduce the along-track velocity components, because of the similar current divisions between segmented collectors and challenges in removing ram velocity, and thus in reliably obtaining ion velocity vectors. IDM also does not operate in low density environments in the dark polar ionosphere and deep density troughs.

In spite of these challenges in measurement technique, precise observations of multi-species flows and non-Maxwellian VDFs are key to understanding the ionosphere-thermosphere-magnetosphere coupling processes.

Specifically, quantitative measurements of flow vectors for all co-existing plasmas are essential to determine the total energy and mass content in the flow and the threshold of instabilities and resulting heating, turbulence, and anomalous resistivity in the ionosphere. It is essential to identify multi-component flow to evaluate the total energy and mass in the flow channels from *in-situ* investigations, since the composition frequently varies in the flow channels^6,7^. Furthermore, inhomogeneity in such flow channels can create free energy by producing perpendicular electric fields and causing plasma heating through instability and wave excitation. For example, ion-ion acoustic instability between H^+^ and O^+^ bulk flows^8,9^, can cause ion acoustic fluctuations^10^. This process is thought to be a major cause of the anomalous resistivity frequently observed by radar under a weak field-aligned current (<100 *μ*A/m^2^)^11^, since acoustic fluctuations are thought to scatter drifting electrons effectively^12^. Moreover, Bergmann and Lotko (1986)^13^ studied the stability of O^+^-H^+^ flows in the ionosphere, finding that two-stream instabilities can be a key heating mechanism for upflowing auroral ions. Sometimes, ion energization and upflows are collocated in the convective flow reversal region^14^, where the extreme ion heating from observations cannot be explained by classical Joule heating alone. Ganguli *et al*.^15^ suggested that even a small amount of velocity shear in the flow is sufficient to excite a large-scale, Kelvin-Helmholtz mode, and can be a potential source for ion heating through wave generation. Recently, Saleem *et al*.^16^ theoretically proved that both ion shear flows and electron field-aligned current (FAC) can produce ion acoustic turbulence, assuming multi-species (O^+^-H^+^) plasmas under realistic ionosphere conditions. Both of these shear-driven mechanisms can contribute to the anomalous resistivity as well.

Ionospheric plasma VDFs frequently show non-thermal features^17,18^. Frictional heating from relative velocities between drifting ions and neutrals produces specific, non-thermal VDFs depending on mass^6,19,20^, which is one of major mechanisms to deposit solar wind energy into the ionosphere^21^. These ion VDFs are studied by past theoretical and numerical predictions, and are predicted to evolve from pancake to torus VDFs depending on the relative speed and mass ratio between ions and neutrals^17,22^. Another mechanism is the resonant heating with waves^23–25^. In the wave resonance process, pronounced transverse heating becomes evident as a noticeable field-aligned asymmetry in VDFs. In such a process, the bulk ion population interacts with waves through cyclotron resonance based on the mass. The Freja satellite observed core ion (<20 eV) bulk heating without any apparent tail-heating feature^26,27^. Sometimes, a fraction of the ion population in the tail near the wave-phase velocity is resonantly accelerated^28^, producing specific non-thermal distributions. Early sounding rocket experiments^29,30^, discovered enhanced ion fluxes in a high-energy tail for pitch angles near 90° at 500 km^24,31^. These transversely accelerated ions (TAI) are mostly mass dependent^32^ and observed at altitudes as low as <500 km by sounding rockets^33–35^, with characteristic energies of > 10 eV. However, most investigations are carried out by short-duration sounding rockets, and continuous satellite observations are still very limited. Also, sounding rocket observations are dominantly triggered by auroral activities^31^. Therefore, 3D VDF measurements at dayside, cusp, polar cap and subauroral regions are still rare; and, even for the aurora, long duration observations are still required for comprehensive understanding.

Ionospheric ions frequently flows upward with a speed up to a few km/s along the magnetic field lines in the polar region^36^. These ions are thought to be the source of the ion outflow by gaining even more energy – enough to escape from the Earth’s gravity. The non-thermal distributions of upflowing cold ions are thought as the initial signature of ion outflow and the driving mechanism can be investigated directly by measuring VDFs. Low-altitude TAIs are one important mechanism to produce upflowing ions with the conservation of adiabatic invariant and can form ion conics at high altitudes^37,38^. FAC or electron precipitation can cause vertical expansion of ambient electrons through heating and the resulting density gradient creates an upward ambipolar electric field. This process can accelerate ions upward along the magnetic field line as well^39–41^. In this case, the VDF of upflowing ions should have a different feature compared to TAIs. The mechanisms involved in the ion outflow and the overall relative importance of each mechanism are still not sufficiently studied and quantified^42^. In addition, it is also known that the ionospheric outflow characteristics depend on season, magnetospheric activity, and even solar cycle phase^43–45^. Further, flow vectors for major ion species can be used to evaluate if the gravity effect can explain the mass dependence of the upflowing processes. By combining the capabilities of the mass analysis and the 3D VDF measurement, physical processes and mechanisms to produce upflowing ions can be directly investigated.

Due to the importance of mass-dependent flows and 3D VDFs observations, a series of instruments have been developed and flown to improve the measurements by RPA type sensors. Dynamics Explorer’s Retarding Ion Mass Spectrometer (RIMS)^46^ used magnets to determine ion composition but could not measure 3D VDFs. Freja’s Three-dimensional Ion Composition Spectrometer (TICS)^47^ used a top-hat ESA^48,49^, with magnets that achieved full-sky coverage using S/C spin. The TICS-type instrument was successfully used on sounding rockets^50^ and identified VDFs of transversely accelerated O^+^ ions^33,51^, but with coarse angular resolutions. Such instruments are ideal for slow-speed rocket observations, but are not optimal for low-Earth orbit (LEO) operations since the fast S/C speed and cold ion temperatures cause the signals to concentrate around the ram direction in phase space. Recent LEO instruments (e.g., Swarm and e-POP) used a HEA^52^, which is ideal for fast-cadence observations. Thermal Ion Imager (TII) on Swarm^53^ combined two, clocked, 2D ion velocity imagers based on the HEA geometry in order to deduce ion temperature anisotropy (T_||_ and T_⊥_); however, it still has to assume 2D Maxwellian VDFs and single species dominance. Swarm was also unable to completely solve the along-track velocity issue^54^ because of the narrow field of view (FOV) to cover only in the perpendicular plane or the parallel plane. When the peaks of velocity distributions are away from these two crisscrossed plains in a velocity space, modeling is required to calculate the density and the bulk velocity direction. The Imaging and Rapid-scanning ion Mass spectrometer (IRM) on e-POP^55^ finally realized mass-resolved, 3D ion VDF detection using HEA, toroidal electrostatic deflector, and gated time-of-flight (TOF) mass spectrometer. However, following issues still exist: (1) coarse energy and angle resolution (inherent to HEA), (2) energy-dependent deflection angle, and (3) significant dead time because the gated TOF causes narrow dynamic range. Table 1 compares the performances of these instruments to 3DI, showing that 3DI could resolve these issues by optimizing performance in the topside ionosphere from LEO, thereby determining ionospheric cold ion properties.

**Table 1:** Comparison of previous ion sensors enabling composition, anisotropy, and non-thermal distribution analyses.

Instrument	TICS	TII	IRM	3DI
Mission	Freja	Swarm	e-POP	TBD
Composition	Yes	No	Yes	Yes
Anisotropy	Yes	Yes	Yes	Yes
D VDFs	Yes	No	Yes	Yes
Angle Res.	12° × 24°	~2.5° × 8°	(3°−45°) × 22.5°	3.2° × (1°−11°)
Energy Res.	10%	5–25%	5–25%	8%

## Methodology

### 3DI design

3DI measures flow velocities, fluxes, and 3D VDFs for major ion species with the angular/energy range and resolution required to resolve the science questions described in the introduction. In addition to the nonthermal features in the VDFs, 3DI can also deduce density, temperature, and flow vector for major ion species in the ionosphere using plasma moments.

**Figure 1:**
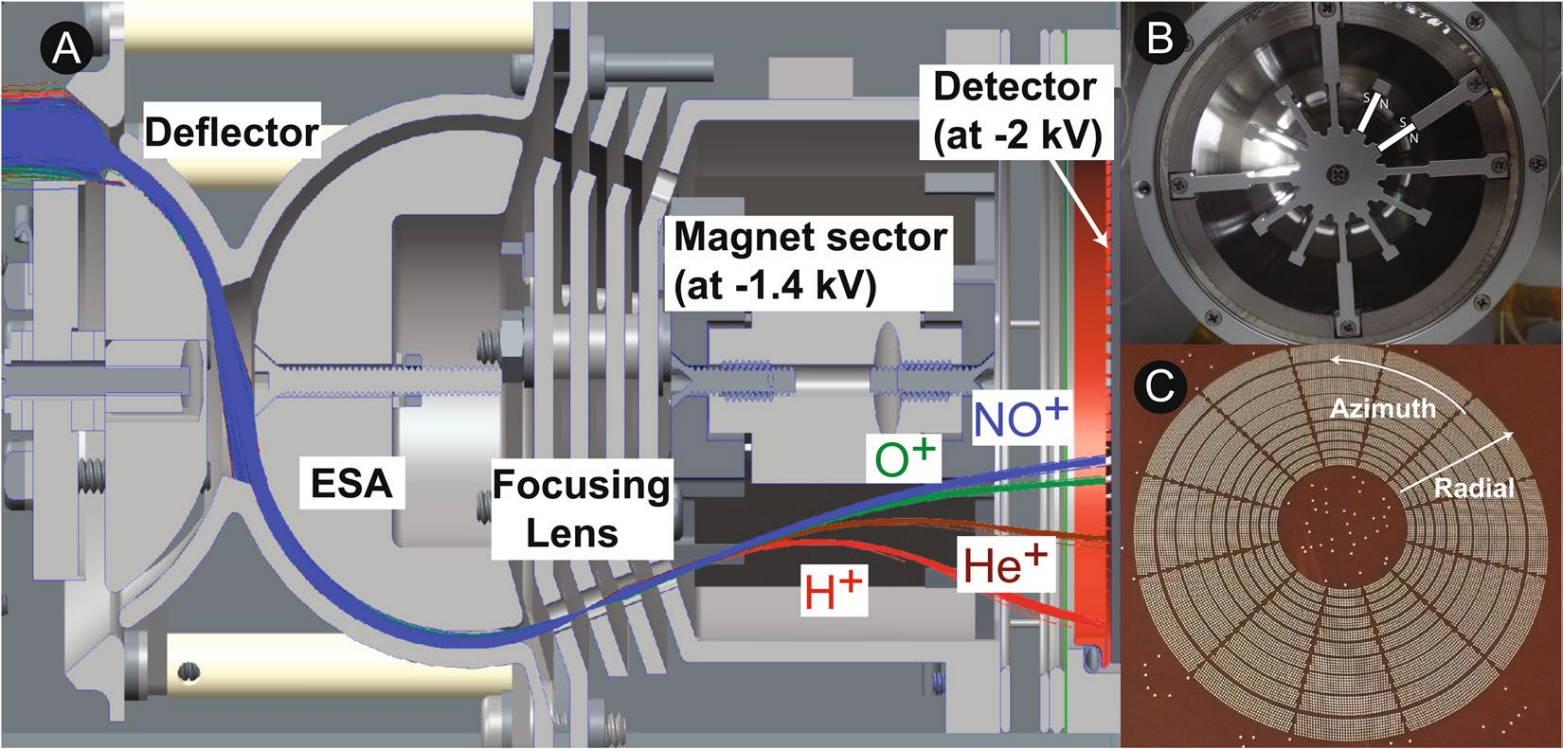
Summary of 3DI design, principle, and performance. (**A**) 3DI cross-sectional view with sample ion trajectories. (**B**) Bottom view (MCP is taken off) of the magnet sector and the high-*μ* shield. The locations of the magnets (two samples out of  twelve cylindrical segments) and their orientations (N or S) are marked in the figure. (**C**) A picture of micro-pixel anodes.

A cross-sectional view of the 3DI mechanical model and sample ion trajectories are shown in Fig. 1. These ion trajectories are calculated with SIMION^56^. The left side of the figure points the ram direction of the spacecraft. The cold ion particles ram into 3DI through the aperture grid depending on the spacecraft velocity.

In the first half of the instrument, the arrival elevation angle and the energy per charge of ions are analyzed. An electrostatic deflector selects the particle arrival directions by applying a positive potential to the outer electrode. The deflector is able to scan the particle arrival directions within the ±32° cone. After passing through the deflector, incident ions enter the ESA section. If the speed of ions is within a certain pass band, ions can transmit through the ESA section without hitting the walls due to the balance of dominant forces: the centrifugal force and the concentric electric force. The 3DI ESA section uses an axi-symmetrical design combining two ESAs back to back. This ESA configuration allows to adapt to modify the sensor FOV to cover near the ram direction with the desired annulus shape and provide a better energy resolution with a longer particle path in the ESA. The entrance ESA, a shorter section with a 30° bend, connects to the exit ESA, a longer 100° bend, through a field-free section. The analyzer constant (the ratio between the applied voltage and the modal energy of transmitting ions) is designed to be the same for both sections, so that the entire ESA can be operated through one power supply voltage applied to the inner curvature electrodes.

Following the ESA, the particles enter a post-acceleration section to gain an additional energy of 1.4 keV so that a constant configuration can be applied for the mass analysis and the detection in the next step. In this section, a set of cylindrical electro-static lenses focuses the ion trajectories and minimize the radial dispersion of ions. These accelerated ions are then deflected in a cylindrical mass analysis sector based on permanent magnets (~0.1 T, NdFeB, total 150 g per 12 segments, Fig. 1B). The magnet sector is also floated by −1.4 kV in order to avoid any additional trajectory deformations on ions. Since the incident energy ranges up to 30 eV per ionic charge for this sensor, the −1.4 kV post acceleration would shrink the dispersion of ion energies to less than the ESA energy acceptance band (8%). The ions are now highly collimated in velocity by the post acceleration. When passing through the magnet sections, their trajectories are bent depending on their mass per charge: lighter ions are deflected more than heavy ions (Fig. 1A). Note that ionospheric ions are mostly in a single ionic charge state; in this configuration the mass spectrometer can resolve the incoming ion mass in principle. Magnets have anti-scattering surface structures consisting of parallel microslats. The magnet configuration and the high-*μ* cover efficiently minimize the fringing field to be 0.1–0.2 Gauss right at the exit of the focusing lens to minimize the effect on low-energy ion trajectories, as confirmed by a magnetometer. Finite element method modeling shows that ~10 cm makes the fringing magnetic field <50 nT from the aperture of the high-*μ* cover. Thus the effect of the magnetic field on the other sensors (if any) aboard the S/C will be very limited or negligible, especially under the relatively high ionospheric magnetic field.

After passing through the magnet sector, the ions are spatially separated based on their mass per charge (in radial direction in Fig. 1C) and their arrival direction (in azimuth direction in Fig. 1C). The position-sensitive detector, MCP, detects these ions efficiently and determines the locations and count rates per each mass/azimuth channel. The surface of the MCP (and grid) is floated around −2 kV to accelerate the ions for higher detection efficiency and simpler read-out electronics by keeping them near the ground potential. After the counts are accumulated in each mass/azimuth channel for a certain time slot, the deflector and the ESA voltages are changed alternately to scan different energies and elevation angles.

### Strategy to cover the ion velocity space

Before describing the laboratory calibration results in detail, this section summarizes how the 3DI instrument scans over the velocity space to obtain phase-space densities of ions. In LEO, the S/C velocity is dominant compared to the bulk or thermal speed of cold ions in the S/C motion frame. In the case that the ambient ions have no bulk velocity component, their velocity is due only to the thermal contribution in VDF. Therefore, ions are detected primarily from the ram direction, and any observed dispersion around the ram direction is due to the thermal velocities. Once the ram velocity vector is subtracted from the measurement, the image of particles spreading within the entire cone angles of 3DI contains information of the type of VDFs (i.e., Maxwellian or non-Maxwellian) and the ion temperature or anisotropic ion temperature (i.e., *T*_||_ and *T*_⊥_) for each species. A Maxwellian distribution would appear azimuthally symmetric (without temperature anisotropy) about the center axis aligned to the ram direction, or line-symmetric about the magnetic field vector orientation (with temperature anisotropy) projected to the sensor FOV. The non-maxwellian distribution would produce either highly asymmetric distributions, or characteristic decay profile in the energy space. Then, for the ions with a certain bulk velocity, the whole distribution of ions will move around in the sensor FOV by the cross-track velocity, or the energy of all particles included in the distribution shifts from the ram energy by the along-track velocity. Thus, the sensor FOV determines the range of the cross-track speed depending on the ion’s bulk energy and the energy coverage limited by the ESA.

**Figure 2:**
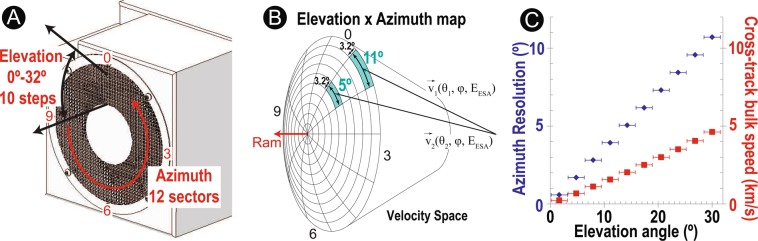
(**A**) 3DI field of view. (**B**) 3DI angular pixel coverage in elevation and azimuth directions with a full deflector sweeping in velocity space. (**C**) 3DI azimuth resolution and cross-track velocity coverage. With 3DI geometry, azimuth resolution is better near the ram.

Figure 2A shows the FOV of 3DI: the actual elevation angle and azimuth angle coverage in the real space. The sensor aperture is expected to point the S/C ram direction in order to maximize the speed-coverage capability. Figure 2B illustrates the coverage of each sector in velocity space with the deflector scan for one ESA voltage step. Instantaneously, 3DI measures a concentric ring with a 3.2° wide in the elevation angle (*θ*) depending on the deflector voltage and the ESA step. One deflector step shall move the ring inward towards the ram direction. By completing one deflector scan, 3DI scans over a part of a sphere in a velocity space limited by the ±32° cone FOV (the mesh surface in Fig. 2B). The ESA scan, which is not described in this figure, varies the radius of this sphere. The azimuth sector (*ϕ*) of 3DI resolves 12 wedges as illustrated on the surface of the sphere. As seen in the figure, one azimuth sector covers 11° wide on the first deflector step (indicated by ${\overrightarrow{v}}_{1}$), as illustrated in the outermost ring in Fig. 2B. On the fifth deflector step (indicated by ${\overrightarrow{v}}_{2}$), the azimuth sector only covers 5° wide in the velocity space because the circumference of the ring gets shorter in the velocity space. In this way, the pixel azimuth resolution varies through deflector steps from 11° to 1°. The amount of this effect is plotted in the blue data points in Fig. 2C. All 12 azimuth sectors measures nearly arond the ram direction in the velocity space on the final (10th) deflector step, and the count rate profile is expected to be similar for all sectors.

The benefit of this configuration is that the bulk ion directions near the ram direction can be resolved with a finer angular resolution. Therefore 3DI is able to determine the along-track component precisely. The dynamic range to cover the cross-track velocity is determined by this angular coverage under a given S/C speed. When assuming typical spacecraft speed (*V*_*SC*_) in LEO (e.g., 7.6 km/s at ~500 km altitude), 3DI covers up to *V*_*SC*_ × sin(32°) = ~4 km/s of the cross-track bulk velocity component with the full FOV (the red data points in Fig. 2C). Considering that ~15° is required for the 2 km/s cross-track speed (Fig. 2C), 3DI can achieve 3.2° × 5° pixel resolution up to ±2 km/s of the cross-track velocity component. On the other hand, the along-track velocity coverage is determined by the ESA operation limit (30 eV per charge). Therefore, the coverage limit is mass dependent. For example, 3DI covers up to ±10 km/s for O^+^ ions and up to ±40 km/s for H^+^ ions. Within the velocity dynamic range (e.g., ±10 km/s along-track speed for O^+^ and ±4 km/s cross-track speed for all species), 3DI can determine the bulk velocity vector independent of the angle from the ram velocity vector. Both along-track and cross-track bulk velocities are calculated as a part of plasma moment. Therefore, the bulk velocity can be determined in sub-pixel or sub-step resolutions. In a typical case (Maxwellian VDF with 400 m/s along-track flow component, 3000 /cc, 1000 K), we obtained parameter errors of 50 m/s (13%), 210 /cc (7%), and 71 K (7%) in one sigma within 1 second accumulation with full energy-angle space scan using a numerical simulation code described later.

## Results

### Performance summary

**Figure 3:**
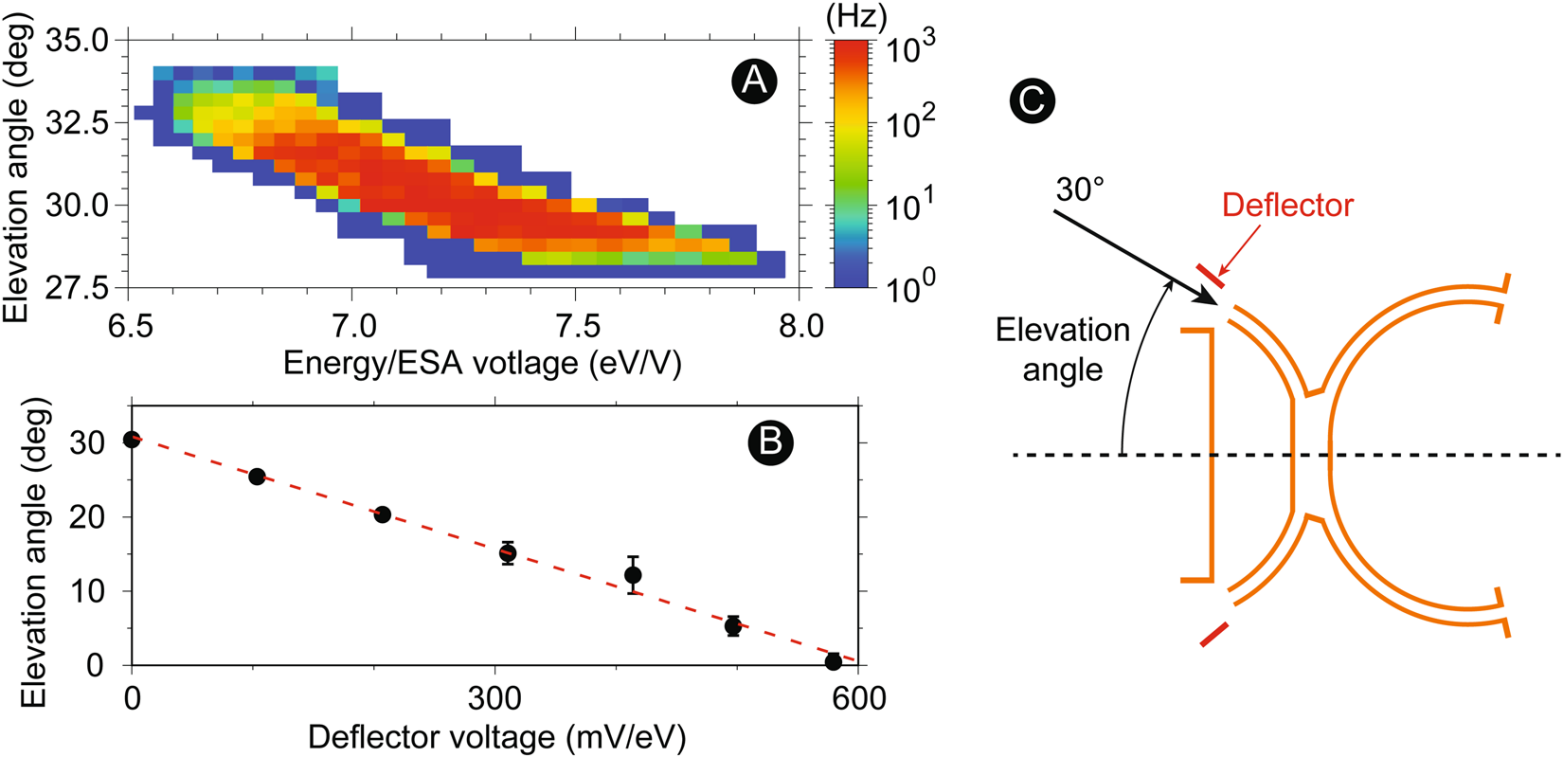
Summary of laboratory calibration results on the deflector/ESA section. (**A**) Energy vs. elevation angle response of the ion transmission rate for 3DI. (**B**) The 3DI deflector performance (deflection angles as a function of applied voltages). (**C**) A schematic drawing of the testing configuration used in this study. The elevation angle and the azimuth sector (around dashed line) were selected by the positioning system in the vacuum chamber.

Table 2 summarizes the 3DI specifications. The elevation-angle step size is ~3.2° (10 deflector steps over 32°) with the instanteneous resolution of 2.6° (cf. Fig. 3A) in full width half maximum (FWHM). Twelve sectors cover the entire azimuthal angle range, with their angular-step size depending on the elevation angle (1°–11°). The ESA resolves the energy per charge of incident ions in 64 voltage steps, with 8% step size (7.5% resolution in FWHM) from 0.1 to 30 eV per ionic charge. The cadence of the instrument is expected to be 1 second in a typical ionospheric plasma density (cf. Fig. 6C) for sufficient counting statistics. In order to meet this cadence, the voltage sweeping scheme is as follows: (1) Deflector is set at one voltage, (2) the energy is stepped through all voltages in every 1.6 ms/step, and (3) Deflector is set to the next voltage. The 3DI’s cylindrical magnet analyzer is optimized to separate H^+^, He^+^, O^+^ (or N^+^), and Heavy ions (NO^+^, ${{\rm{O}}}_{2}^{+}$, and ${{\rm{N}}}_{2}^{+}$) which are the primary cold ion species in the ionosphere. Proper assumptions or models are required to separate between O^+^ and N^+^ or among heavy ions. For example, O^+^ and NO^+^ are dominated species in the fast flow channels, which are related to most of the science goals of 3DI, both from observation^5^ and modeling^6^. The 3DI flight electronics is based on the Suprathermal Ion Sensor (SIS) instrument^57^ qualified and delivered for the CubeSat Mission for studying Solar Particles (CUSP) mission^58^ to be launched in ~2021. Sensor volume, mass, and power estimates in the table are based on a typical example of the 3DI flight configuration. The data rate is assumed to detect full sky ion velocity distributions in 1 s cadence without using compression. In this calculation, the instrument duty ratio is assumed to be 39% (>55° around the north pole, and <−55° around the south pole).

**Table 2:** 3DI specifications.

Geometric Factor (total)	3.5 × 10^−6^ cm^2^ str eV/eV
Energy Range	0.1–30 eV
Energy Resolution (ΔE/E)	8%
Energy steps	64
Time resolution	1 second
Angle Coverage (with deflector)	Ram ± 32°
Angle resolution (elevation)	3.2° step (2.6° in FWHM)
Angle resolution (azimuth)	1°−11° step
Target ion species	H^+^, He^+^, O^+^ or N^+^, and Heavy ions
Sensor Mass	1860 g
Sensor Volume	1.9 U
Power	4160 mW
Data rate (before compression)	307 kbps

**Figure 4:**
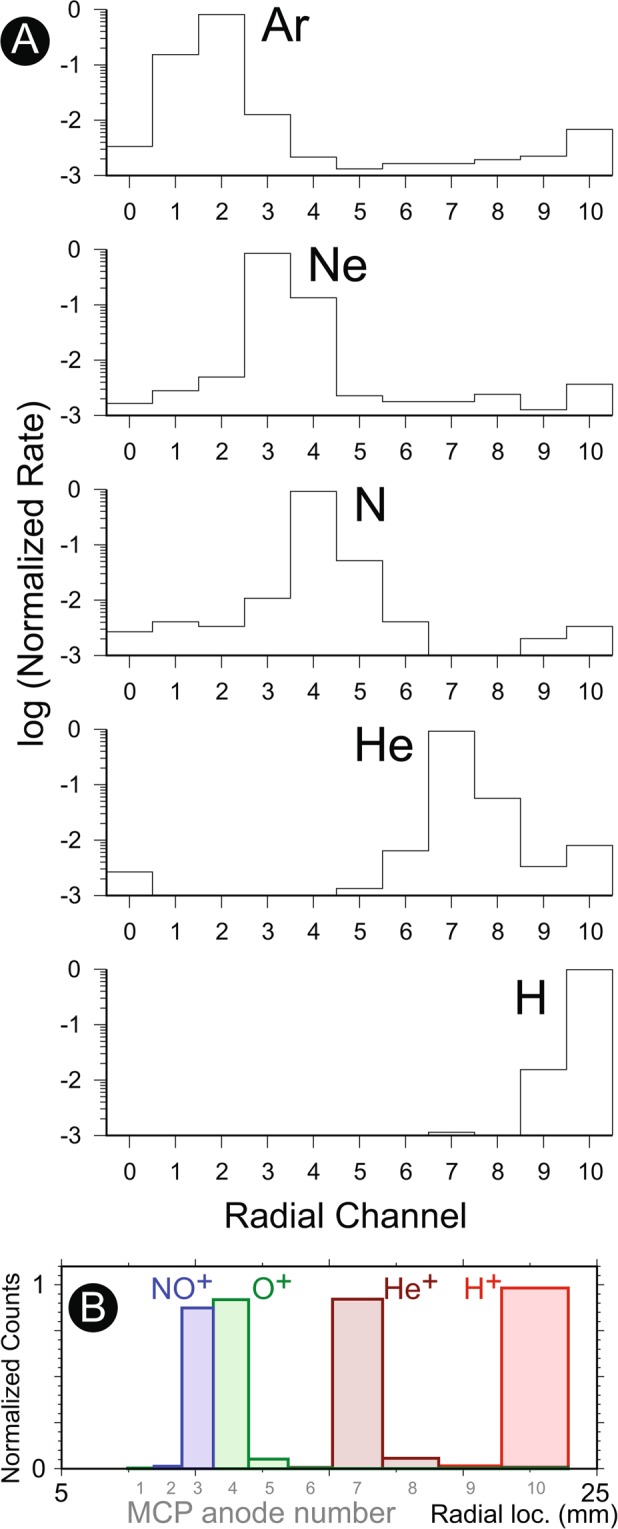
A summary of 3DI mass spectrometer performance. (**A**) The logarithmic count-rate distributions for each anode using ions with seven different mass numbers. The axis of abscissa shows the anode number from inside to the outside (The center hole in Fig. 1C was filled with the 11th anode for the testing configuration, as indicated by the anode number zero). Given that the speed of ions is nearly identical due to the post acceleration, the ion mass disperses the landing location with a cylindrical magnetic field sector. (**B**) Expected mass separation capability of 3DI for four major ionospheric ions.

**Figure 5:**
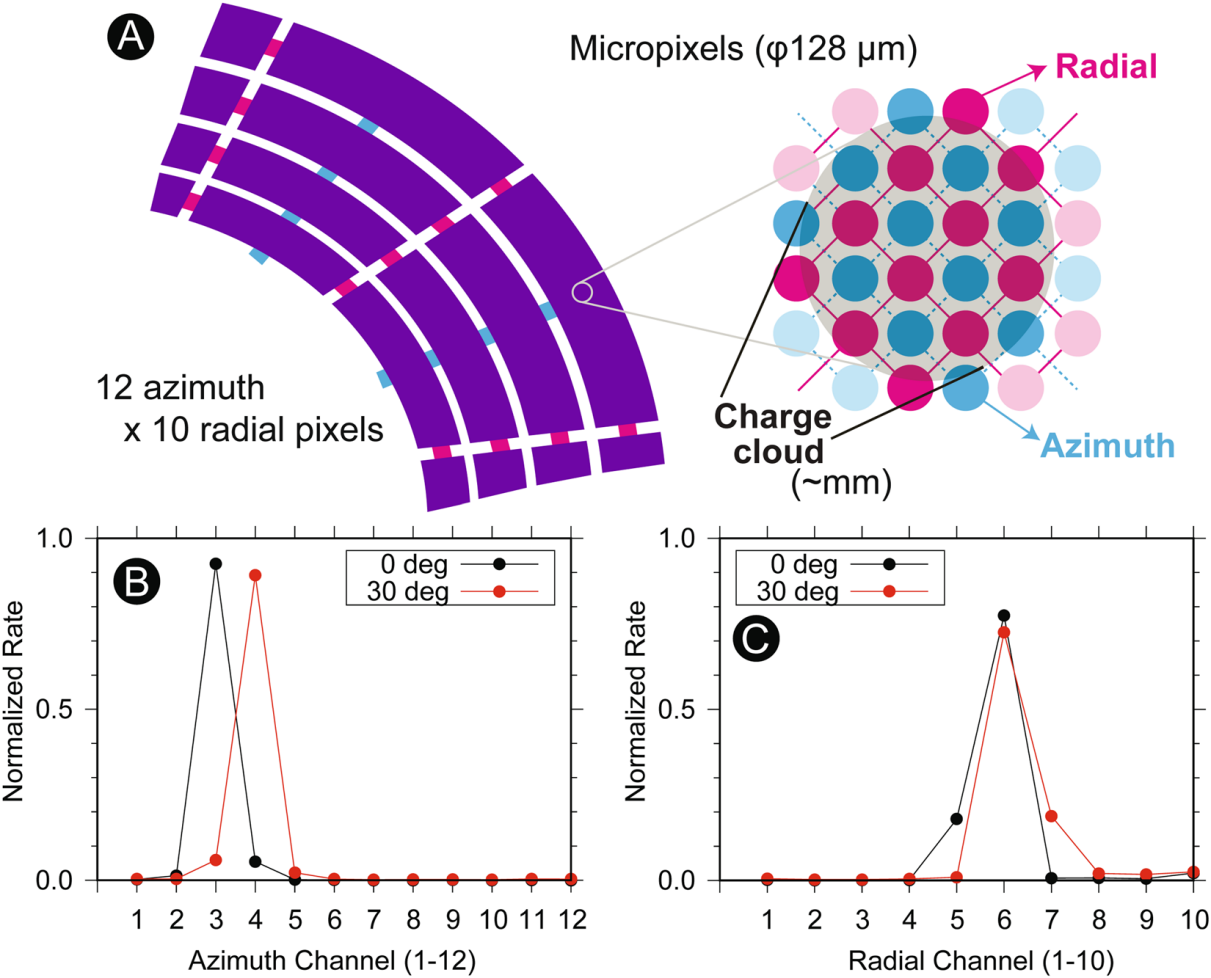
(**A**) A schematic of the operation principle of the micro-pixel anode. (**B**) The micro-pixel anode performance for an azimuth chain. (**C**) The micro-pixel anode performance for a radial chain. The figures labeled B and C were taken simultaneously.

**Figure 6:**
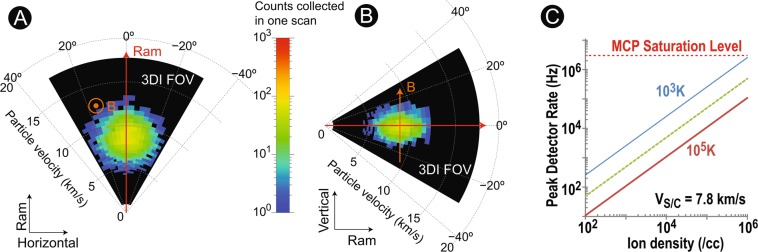
A 3D anisotropic VDF within a (**A**) horizontal plane (3–9 in Fig. 2A) and a (**B**) vertical plane (0–6 in Fig. 2A) for a typical O^+^ ion flow in the ionosphere. (**C**) An estimated count-rate performance of 3DI under a typical range of ions in the topside ionosphere.

### Proof of concept study

The ESA system and the magnet mass spectrometer system were investigated and the performance and functionality of the 3DI prototype were investigated. The prototype 3DI was set in a vacuum chamber and mounted on a 4-axis, computer-controlled positioning system that allowed us to orient the aperture of the sensor with respect to the incident ion beam. The ion source was connected to the chamber via a few-meter flight tube. In order to achieve a fairly uniform intensity of the beam, the source beam energy was set <5 keV depending on the species, then decelerated to a relevant energy range. The ion species are selected using a permanent magnet right before the entrance of the flight tube. For this purpose, the entire beam system and the controller were electrically floated at the negative voltage with a set of insulators along the flight tube. For the count processing and accumulation in a certain modifiable cadence, a flight-ready electronics boards identical to SIS^57^ was integrated into the prototype. During the measurement, a beam monitor was used to check the uniformity and stability of the beam before and after a measurement sequence.

### Electrostatic analyzer

Figure 3 summarizes the 3DI ESA performance confirmed in the laboratory. The measurements were obtained using 500-eV Helium beam without applying the deflector voltage. This energy was chosen for the beam stability for Helium. Although the beam energy is higher than the expected energy range, the electro-static performance of the system can be evaluated by scaling the electrode voltages up. For the flight operation in the low-energy range, the stability of the high-voltage power supply was confirmed in the required voltage range. When the energy step is needed for evaluation purposes, the ESA voltage was varied instead of the beam energy to keep the beam emission constant during the measurement sequence. This technique is commonly applied (energy scaling and voltage scanning) for the calibration of space-plasma particle instrumentation.

Figure 3A shows an ESA transmission property as a function of the incident ion energy (normalized by the ESA voltage) and the elevation angle for one azimuth segment. The transmission of the ESA was evaluated by the total count rate on one detector segment relevant to the incident azimuth sector. The angle and the voltage were scanned with 0.4° and 1 V steps. No deflector voltage was given during this measurement, thus the elevation angle representing the highest transmission was expected to be around 30° because the particles inject along the aperture of the entrance ESA (Fig. 3C). This figure represents the trend of the accepting energy range as a function of the incident elevation angle. For 3DI, the higher-energy particles tend to go through the ESA when they have a slightly slanted incident direction towards the ram. Even though two ESA segments are combined back to back, this trend preserves the properties of the spherical or top-hat analyzer. Based on the laboratory measurement, the intrinsic angular resolution was 2.6°, the energy resolution was 7.5%, and the analyzer constant was 7.17 (all in FWHM).

The deflector performance was also tested for all expected arrival angles and plotted as a function of the applied voltage (mV/eV) in Fig. 3B with the similar beam condition as the ESA calibration. For this testing, the ESA voltage was fixed, and only the elevation angle was scanned by 0.4° step and investigated from 0° to 30° at seven deflector voltages. The data point shows the modal value for the transmission distributions as a function of the beam angle (representing the elevation angle). The error bar in Fig. 3B reflects one-σ errors obtained by Gaussian fitting to the measured transmission profile as a function of the elevation angle for each ESA-deflector combination. As clearly seen in the figure, the elevation angles linearly responded to the applied voltage. This deflector system requires ~20 mV/(eV/q) per 1°. In order to operate 3DI in the full required FOV range (30° deflection for 30 eV/q), ~18 V is required as the maximum deflector voltage. The angular resolution was <3° for most of the range, deteriorating slightly to ~5° for 35–45°. In order to avoid secondary electron emissions from the deflector electrode by UV illumination in space, only the positive voltage will be applied to deflect particles for flight operation.

### Mass spectrometer

Figure 4A shows the result of mass spectrometer operations using H, He, N, Ne, and Ar ions. In this testing, the ion energy was adjusted to reach 1.4 keV after the post acceleration (thus, the post acceleration voltage was ~−900 V total with 500 eV beam). As seen in the numerical simulation (Fig. 1A), the lighter ions exhibit a significant bend toward the outer channels due to the tangential magnetic field, while the heavier ions almost free-fly toward the center channels. The landing locations of ions reflects this behavior, and they are distributed with distinctive peaks based on their mass. The peaks are located at channel 10 for H^+^, 7 for He^+^, 4 for N^+^, 3 for Ne^+^, and 2 for Ar^+^. These results clearly demonstrate that the expected mass range (1–40) was adequately covered by the 11 anodes distributed for MCP read-out. Note that these anode widths and locations are not evenly but logarithmically separated since higher spatial resolution is required for heavier species, especially O^+^ and NO^+^. The counts distributed around the peak channels cause a slight crosstalk and ambiguity of the mass analysis. Based on these measurements, the contamination rate from He ion counts to the H ion channel was 1.1%, and the contamination rate from H ion rate to the He ion channel was 0.2%. The contamination rate from He ion rate to the N ion channel was 0.3%, and the contamination rate from N ion counts to the He ion channel was 0.5%. In the actual topside ionosphere, the most dominant ion species are H^+^, He^+^, O^+^, and NO^+^ and the 3DI goal is a reasonable separation of these ions. Thus the performance for these four species is important and worth evaluating. Unfortunately, our beam cannot produce clean O^+^ or NO^+^ beams in the required energy range. Instead, the 3DI’s mass separation capabilities for O^+^ (16 AMU) and NO^+^ (30 AMU) are interpolated based on these laboratory measurements by N^+^ (14 AMU), Ne + (20 AMU), and Ar + (40 AMU). Figure 4B shows the expected H^+^, He^+^, and O^+^, NO^+^ count rate distributions assuming the same normalized rate for all species. This figure also shows the locations of the anodes in the real space in addition to the anode numbers. The calculated cross-contamination rate shows that the highest cross contamination, between O^+^ and NO^+^ channels, was 1.4%.

Persistent counts for the outer anodes in the <1% level are produced by the hydrogen originating from the residual water gas in the beam source. Also, these water ions might have caused a slight mixture of the counts around N and Ne by producing OH^+^ and O^+^. A more precise evaluation of these contamination sources would be beneficial for flight-model calibration, however such efforts are beyond the scope of this paper since our results suggest that the basic operation principle and the performance capability required for science are sufficiently demonstrated.

Lower beam energy might also be available with a minor modification on the beam source (use single species gas rather than the mixture gas used for the testing and get rid of the magnetic mass filter), and/or the direct mount of the beam to the chamber without a few-meter long flight tube. Essentially, however, the lower energy ions behave better than the higher energy ions due to the dominance of the post acceleration over the energy acceptance of ESA from numerical simulations. Such low-energy beam evaluations are beyond the scope of the paper for the proof-of-concept study.

### Micro-pixel anode

Signals from the MCP are detected by a novel, micro-pixel anode system (Fig. 5A, also see Fig. 1C for a photograph). The micro-pixel anode is designed to reasonably reduce the number of read-out chains for low-power operation, while minimizing the issues of typical imaging MCP anodes^59^ such as the dead time (delay-line anode system) and the low-pulse height (wedge-and-strip anode system). The design concept of micro-pixel anode is similar to that of cross strip anode^60^: One charge cloud will be shared with several discrete channels to determine the position. However, instead of using the center of the charge distribution for a 2D geometry and fine imaging, the micro-pixel anode connects all micro pixels in a certain area to two read-out chains. This concept allows more flexible shape of the anode segments while providing decent size of the signal charge (divided just in half for 3DI case). Each micro pixel consists of a miniaturized through hole with a 128 *μ*m diameter in a multi-layered anode board. Their locations are both electronically and capacitively isolated to each other. The signal traces in each layer of the anode board are separated from other chains enough so that the capacitive coupling is negligible internally as well. In the 3DI multi-layer board, micro pixels are connected to either one of 10 radial chains or one of 12 azimuth chains (Fig. 5A). One charge cloud from the MCP will hit many of such micro pixels (dark shadow in Fig. 5A), and the signal charge is shared by one chain for radial and one chain for azimuth. Thus, if we take coincidence of detected signals between the radial and azimuth chains, the Field-programmable gate array (FPGA) logic can identify the location (the radial and azimuth chain numbers) and determine the mass and azimuth angle at the same time. By using the 3DI configuration, only 22 read-out chains need to be implemented to determine the triggered location from 120 (10 × 12) pixels while the signal strength and the response time are almost equivalent to the direct read-out method such as multi-anode system.

Figure 5B,C summarize the micro-pixel anode performances by comparing two configurations (represented by black and red plots). The identical beam was used for both configurations, while only the incident azimuth sector was shifted by 30° to the adjacent azimuthal wedge around the instrument symmetric axis. In response to the rotation of the instrument, the active channel moved from channel 3 to channel 4 (Fig. 5B). At the next wedge, as shown in Fig. 5C, the count rate profile did not change significantly, with a clear peak at channel 6 for both cases because the ion mass remained essentially identical. The cross talk between pixels (multiple detection) was almost negligible during the measurement. The slight broadening of the distributions (extending from channel 3 to 4 and channel 4 to 3 in Fig. 5B) is the beam size effect that stimulates adjacent channels at the same time, while the difference of the tail direction in Fig. 5C is caused by the non-uniformity of the magnet strength throughout the wedges.

### Sensor performance modeling

Figure 6A,B model the performance of 3DI under typical topside ionosphere conditions by assuming realistic ion parameters and S/C velocity. The O^+^ ion flow is assumed with a density of 10^4^/cc and a bulks speed of 8 km/s (including the S/C ram speed). For the source-ion VDF, a temperature anisotropy of ~3 (T_||_ = 1000 K, and T_⊥_ = 3000 K) is assumed when the O^+^ ions are heated perpendicularly in the polar cap. For 3DI, the 64 energy steps, 10 deflector steps, and instrument sensitivity (detector efficiency of ~50%) are considered to calculate the counts to be accumulated in one bin when operating the instrument in a 1 s cadence (Table 2). Figure 6A shows a 2D cut of the full 3D distribution in the horizontal direction. In the polar ionosphere, the terrestrial magnetic field can be nearly perpendicular to the plane, as indicated in the figure. The whole ion VDF is observed as a shifted Maxwellian distribution by the ion bulk velocity (8 km/s) to the ram direction. For the vertical cut (Fig. 6B), a squashed VDF is observed along the field line due to the anisotropy assumed in the calculation. From this calculation, the target ion anisotropy (~3) is clearly detectable within the resolution, FOV, and dynamic range of 3DI. Figure 6C shows the estimated count rate of 3DI under typical ionospheric conditions (density and temperature). The 3DI design is optimized for densities of 10^2^ to 10^6^, with temperatures between 10^3^ to 10^5^ K. Within this range, the MCP can be operated normally without experiencing serious saturation.

## Discussion

The investigations enabled by 3DI are extremely useful in three active and critical areas for current/future Heliophysics research.

The inclusion of ion temperature anisotropy in the ionospheric numerical models is essential for the accurate prediction of thermal environment and ionosphere structure, though most ionospheric models use isotropic, collisional fluid descriptions. Under the strong perpendicular accelerations, especially for ion-neutral collisions, the parallel temperature is usually overestimated, therefore the scale height may contain a significant error since the magnetic field is almost vertical in the polar region^6^. Recent new modeling efforts, including the anisotropic fluid model^61^, suggest that the isotropic model can overestimate field-aligned, ion-velocity responses by as much as ~48% due to the effects of ionospheric upflow driven by DC electric fields. Moreover, climatological models and global simulations with coarse-grid resolution cannot capture composition and heating processes in localized and dynamically evolving flow channels. *In-situ* and comprehensive observations of ion temperature anisotropy accelerate these efforts by providing the ground-truth, *in-situ* observations.

Precise observations of ion anisotropy and compositions have a huge impact on the ground-based, incoherent-scattering radar modeling as well. Such strong bulk ion upflows have also been observed by the European Incoherent Scatter Scientific Association (EISCAT) in the auroral topside ionosphere^62–64^. Associated with these high-speed flow channels, ion heating is frequently observed in the F-region and has been extensively studied with radars^65–67^. Specifically, the ion heating process is thought to be important due to the connection to the ion upwelling process^68^. However, (1) radars do not provide composition without assumptions^69^, and (2) departure from isotropic Maxwellian VDFs toward anisotropic, toroidal, or kappa VDFs is a major source of error in estimating plasma parameters, especially during periods of intense ion heating^70–74^. A recent study applies analytic functions to deduce correct measurement during strong convection^20^, and the *in-situ* verification of ion composition and nonthermal VDFs advance these efforts significantly.

The fundamental process of ion outflow has particularly important implications for the atmospheric loss. Moreover, the properties of outflowing ions are also important for the understanding of the physics associated with geomagnetic storms and magnetic reconnection. Ionospheric ions are thought to be a major contributor to plasma pressure and current throughout the magnetosphere especially during geomagnetic storms. These ions carry energy and mass between ionosphere and magnetosphere, and the magnitude of ion outflow is not only related to the storm strength but also contributes to the storm strength. In addition, the outflowing ions often consist of ions heavier than atomic hydrogen (e.g., oxygen (O^+^) ions). The increase of the ionospheric ions can change the characteristics of the magnetospheric plasma due to the enhanced local-mass density: particle loss properties^75^, particle injection properties^76^, and convection electric field^77^ in the ring current; reconnection rate^78^, location^79^, and shape^80^ in/of the magnetospheric current sheet on dayside magnetopause or in the plasma sheet.

## Concluding remarks

Accurate observations of the mass-resolved ion flow structure and the ion 3D VDFs in the topside ionosphere are extremely valuable, benefiting the Heliophysics community by answering questions that still remain to reveal magnetosphere-ionosphere-atmosphere coupling mechanisms. This paper proposes a novel sensor, 3DI, that can potentially make such observations in Earth’s ionosphere. The prototype 3DI successfully went through proof-of-concept studies in the laboratory for all major components. The sensor performance in the LEO flight is also evaluated using a numerical modeling. The 3DI instrument is designed to fit in a very small form factor (especially for nano satellites) and offers a variety of applications in future LEO missions.
